# Exploring the effects of peri-partum ingestion of traditional medicine on maternal and foetal outcomes: a prospective cohort study

**DOI:** 10.1186/s13104-019-4199-y

**Published:** 2019-03-25

**Authors:** Julius Sama Dohbit, Esther Meka, Joel Noutakdie Tochie, Myriam Mbia Koudo Ze, Felix Essiben, Valirie Ndip Agbor, Jan Rene Nkeck, Pascal Foumane

**Affiliations:** 1 Departement of Gynaecology and Obstetrics, Yaoundé Gynaeco-Obstetrics and Paediatric Hospital, Yaoundé, Cameroon; 2 Departement of Gynaecology and Obstetrics, Faculty of Medicine and Biomedical Sciences, University of Yaoundé 1, Yaoundé, Cameroon; 3 Departement of Surgery and Specialities, Faculty of Medicine and Biomedical Sciences, University of Yaoundé 1, Yaoundé, Cameroon; 4 Ibal Sub-divisional Hospital, Oku, North West Region Cameroon; 5 Department of Clinical Research, Health Education and Research Organisation (HERO), Buea, Cameroon; 6 Departement of Internal Medicine, Faculty of Medicine and Biomedical Sciences, University of Yaoundé 1, Yaoundé, Cameroon

**Keywords:** Traditional medicine, Labour, Maternal, Foetal, Outcome

## Abstract

**Objective:**

In Africa, 80% of women ingest traditional medicine (TM) during pregnancy. Although widely used in Cameroon, no study in has either demonstrated its safety or effectiveness. Hence, we sought to determine the effects of TM ingestions during the peri-partum period on maternal and foetal outcomes. A cohort study was conducted from January to April 2016 in two referral maternity departments of Cameroon. We consecutively enrolled all consenting parturients with gestational age above 28 weeks. We divided them into two groups; exposed and unexposed. The exposure studied was ingestion of TM within 72 h prior to delivery. Variables studied were socio-demographic characteristics, type and frequency of TM ingested and details of labour.

**Results:**

We enrolled a total of 603 parturients of whom 147 in the exposed group and 456 in the non-exposed group. The most frequently used TM were honey and *Triumfetta pentandra A*. Ingestion of TM in the peri-paritum period was associated with intra-partum vaginal bleeding, dystocic labour, tachysystole and uterine atony. No adverse neonatal outcome was observed. Overall, these findings could help guide the direction of future research into the safety and potential benefits of peri-partum TM use, as well as serving as a preliminary reference for counselling.

## Introduction

Traditional medicine (TM) is the sum of all knowledge, skills and practices that are based on the theories, beliefs and experiences of health preservation, specific to different cultures, whether explainable or not, which are used in health preservation, as well as in the prevention, diagnosis, improvement or treatment of physical or mental illness [1]. In TM, drugs of the traditional pharmacopoeia, called “herbal medicines” are used. As high as 80% of pregnant African women in Africa and the diaspora use TM to treat pregnancy related symptoms [2]. According to a study conducted in 2012 in Cameroon, 31.02% of women use TM during the second half of pregnancy [3]. However, its oral intake during pregnancy may be associated with obstetrical, foetal and neonatal complications. A study conducted in South Africa reported that the use of TM increased the rates of emergency cesarean and foetal distress [4]. There is a dearth of data on the impact of TM ingestion in the peri-partum period in Cameroon. Against this background, we conducted this study to determine the effects of the ingestion of TM during the peri-partum period on the maternal and foetal outcomes.

## Main text

### Methods

#### Study design, setting and participants

This was a prospective cohort study conducted from January 1 to April 30, 2016, in the maternity wards of two referral hospitals in Cameroon; the Gyneco-obstetrics and Pediatric Hospital of Yaoundé and the Central Hospital of Yaoundé. An exposed group (consisting of pregnant women who had ingested TM within 72 h prior to delivery or during labour) was compared to an unexposed group (pregnant women who had not ingested TM within 72 h prior to delivery or during labour). We excluded all parturients with a gestational age less than 28 weeks, those who did not consent to participate to the study, parturients in whom labour was induced, those with intrauterine fetal death prior to the onset of labour, those with multiple gestations, those who delivered before hospital admission and those undergoing elective cesarean section. The sampling method was exhaustive and consecutive. Assuming a 95% confidence interval (CI), a variability of 1.96, an accuracy of 0.05, 31.02% rate of TM use [3], the SCHULZ and GRIMES formula was used to obtain a minimum size of our sample of 89 subjects per group. The sampling method was exhaustive and consecutive.

#### Data collection

A pre-tested questionnaire was used to collect information on the socio-demographic characteristics (age, level of education, and marital status), TM details (type of TM ingested, frequency of intake and amount), labour details (reason for admission, duration of the second period of labour, use of oxytocins, complications during labour), delivery details (mode of delivery, APGAR score at the 1st and 5th min, delivery complications such as acute foetal distress and uterine atony).

### Management of delivery

All deliveries occurred with women lying in the recumbent position with legs in holders. Fetal heart monitoring during delivery was done electronically by means of a cardiotocography machine.

#### Definition of terms

Foetal distress was defined as the occurrence of late or variable decelerations and reduce variability of foetal heart rate (less than 110 beats/min) [5, 6]. Blood loss was estimated and postpartum haemorrhage defined as an estimated blood loss greater than 500 mL within 24 h after vaginal delivery or greater than 1000 ml within 24 h of cesarean section [7, 8]. Dystocia was defined as slow or difficult labour related to inefficient uterine contractions, failure to progress, prolonged labour > 12 h, arrested descent and cephalopelvic disproportion [9, 10]. Uterine atony was defined as failure of the uterus to contract after delivery [11] while tachysystole was defined as more than five uterine contractions in 10 min for at least 30 min [12]. Neonatal asphyxia was defined based on the Modified Sarnat-Sarnat Score [13, 14].

#### Data analysis and management

The data was collected, recorded and analyzed using Epi-info 3.5.4 software. The categorical variables were compared using the Chi square test and the Fisher exact test when appropriate. The factors associated with the use of TM were identified by calculating the risk ratio (RR) with its 95% confidence interval (CI). The association between the use of TM and different variables was measured using the relative risks (RR) and its 95% confidence interval (CI). Logistic regression was performed for all variables whose p value was < 0.1.

### Results

#### Incidence and reasons for ingestion of traditional medicine during peri-partum period

A total of 603 parturients met our inclusion criteria. One hundred and forty-seven (147) took TM 72 h preceding delivery or during labour (exposed group) and 456 who did not (the non-exposed group). Hence, the incidence rate of TM intake during the peri-partum period was 24.4%. The types of TM used by parturients are summarized in Table 1. The most frequently used TM was honey in 28.2% and *Triumfetta pentandra A*. (“nkui”) in 23.7% of the cases. Several parturients used a combination of TM. The main reasons for the ingestion of TM were to ease delivery (83.3%), to induce labour (21.36%) or to treat constipation (10%).

**Table 1:** Distribution of the list of Traditional Medicine used in terms of percentage use; reason for using and subjective perception

Traditional medicine	Number (%)	Most reported reason for its intake	Subjective perception
Honey	41 (28.2%)	Ease labour	Very effective
*Triumfetta pentandra A.* (nkui)	35 (23.7%)	Ease delivery	Very effective
Hibiscus leaves (*Hibiscus rosa*-*sinensis*)	29 (20%)	Ease delivery	Very effective
Brimstone tree leaves (*Morinda lucida*)	26 (17.7%)	To induce labour	Very effective
Lemon grass (*Cymbopogon citratus*)	17 (11.4%)	Ease delivery	Very effective
Wild mango (*Irvingia gabonensis)*	15 (10%)	Manage constipation	Very effective
*Vernonia conferta* Benth. Barks	9 (3.4%)	Relieve chronic pelvic pains	Very effective
Unknown herbs	18 (12.3%)	Unknown	Unknown

#### General characteristics of the study population

The ages of the parturients ranged from 15 to 45 years with an average of 28.88 ± 6.31 years in the exposed group and 27.90 ± 6.30 years in the non-exposed group The most represented age group was 25–34 years old. Table 2 shows the distribution of the population by age. With regards to marital status, 48.3% were singled and 36.5% married. Cohabitation was associated with TM ingestion during the peri-partum period (RR = 1.79, 95% CI 1.10–2.94, p = 0.016).

**Table 2:** General characteristics of the study population

Variables	Total N = 603 (%)	Exposed group N = 147 (%)	Non-exposed group N = 456 (%)	Risk ratio (95% CI)	P value
*Age (years)*					
15–25	181 (30)	38 (25.9)	143 (31.4)	0.76 (0.50–1.16)	0.122
26–35	181 (30)	79 (56.5)	238 (57.2)	1.06 (0.73–1.54)	0.407
36–45	105 (17.3)	30 (20.6)	75 (16.4)	1.30 (0.81–2.09)	0.164
*Marital status*					
Single	291 (48.3)	64 (43.5)	227 (49.8)	0.78 (0.53–1.13)	0.111
Married	220 (36.5)	52 (35.4)	168 (36.8)	0.94 (0.63–1.38)	0.413
Divorced	7 (1.2)	2 (1.4)	5 (1.1)	1.24 (0.24–6.48)	0.540
Widow	19 (0.2)	0 (0)	1 (0.2)	0	0.756
Liberal unions	84 (13.8)	29 (19.7)	55 (12.1)	1.79 (1.10–2.94)	0.016
*Level of education*					
No formal education	30 (5)	9 (6.1)	21 (4.6)	1.35 (0.60–0.29)	0.294
Primary	94 (15.6)	20 (13.6)	74 (16.2)	0.81 (0.48–1.39)	0.267
Secondary	277 (45.9)	57 (38.8)	220 (48.2)	0.68 (0.47–0.99)	0.028
Tertiary	202 (33.5)	61 (41.5)	141 (30.9)	1.58 (1.08–2.33)	0.012

Having at a secondary level of education was associated with not using TM prior to labour onset (RR = 0.68, 95% CI 0.47–0.99, p = 0.028). In contrast, having a tertiary level of education was associated with TM oral intake in the peri-partum period (RR = 1.58 95% CI 1.08–2.33, p = 0.012).

#### The influence of traditional medicine on labour

The most common reason for admission was labour pains in 57.4%. TM ingestion in the peri-partum period was associated with intra-partum vaginal bleeding (RR = 1.51, 95% CI = 1.10–2.08, p = 0.011) and dystocic labour (RR = 1.45, 95% CI = 1.10–1.91, p = 0.007). The estimated vaginal blood lost in all parturients did not meet our criteria for postpartum haemorrhage as defined in the methods. Furthermore, TM ingestion during labour was significantly associated with tachysystole (RR = 1.27, 95% CI = 1.06–1.52, p = 0.008) and uterine atony (RR = 7.24, 95% CI = 1.90–27.63, p = 0.002). All uterine atony observed were associated with excessive blood loss. There was no significant association between neonatal complications and TM intake as seen in Table 3.

**Table 3:** Logistic regression analysis of the influence of traditional medicine on labour and delivery

Variables	Total N = 603 (%)	Exposed group N = 147 (%)	Non-exposed group N = 456 (%)	Risk ratio, (95% CI)	P value
*Presenting complaints*					
Lost of liquor	88 (14.6)	15 (10.2)	73 (16)	0.66 (0.41–1.08)	0.051
Vaginal bleeding	100 (16.6)	34 (23.1)	66 (14.5)	1.51 (1.10–2.08)	0.011
Labour pains	346 (57.4)	90 (61.2)	256 (56.1)	1.17 (0.88–1.57)	0.161
APGAR score at 1st min < 7	94 (14.4)	20 (13.4)	74 (14.7)	0.90 (0.59–1.37)	0.367
APGAR score at 5th min < 7	71 (10.9)	13 (8.6)	58 (11.6)	0.77 (0.46–1.28)	0.187
Dystocic labour	160 (26.5)	51 (34)	109 (23.9)	1.45 (1.10–1.91)	0.007
Tachysystole	279 (46.3)	81 (55.1)	198 (43.4)	1.27 (1.06–1.52)	0.008
Uterine atony	10 (1.7)	7 (4.8)	3 (0.7)	7.24 (1.90–27.63)	0.002

### Discussion

This study aimed to determine the effects of TM oral intake during the peri-partum period on the maternal and foetal outcomes. We found that the ingestion TM within 72 h prior to delivery or during labour was significantly associated with vaginal bleeding prior to admission, tachysystole, dystocic delivery and uterine atony. TM use during the peri-partum period had no effect on foetal outcome.

#### Incidence of TM oral intake during labour and general characteristics of the study population

The incidence of TM ingestion in this study was 24.4%. This incidence is lower than that of Holst et al. in Sweden [15], Mabina et al. in South Africa [16] and Fakeye TO et al. in Nigeria [17] who observed incidence rates of use of TM of 36%, 55% and 67.5% respectively. This disparity could be explained by the fact that their incidences entail the use of TM during pregnancy and labour, compared to our study which focused on peri-partum use only. About eight different TM were reported to be ingested by parturients in our cohort, of which the most common were honey (28.2%), *Triumfetta pentandra* A. (23.7%), followed by the Hibiscus rosa-sinensis L (20%). Several parturients reported to use more than one TM during labour. This result is similar to that of Forster DA et al. in 2006 [18], who found an average TM combination of two per women. Congruent with the findings of Awouda et al. [3] and Azriani et al. [4], the main reason for TM consumption during labour was to ease delivery. The most represented age group was aged between 25–34 years. This is similar to the report of Hepner et al. in Massachusetts [19]; suggesting that the 25–34 years age group might be a potential target group to implement preventive strategies for TM use during pregnancy and labour.

Cohabitation was associated with TM ingestion prior to delivery or during labour. Although we could not find a plausible reason for this association, our results corroborate with those of Kofi AS in Ghana [20] and Fakeye TO et al. in Nigeria [21].

Secondary education was found to convey protection against TM use while tertiary education increased the odds of herbs intake during the peri-partum period. These results concur with those of several authors [15, 22–25]. This can be explained by the fact that women with a lower level of education are more likely to rely on the recommendations of their health care providers who do not often recommend TM use during gestation because of their unknown clinical efficacy or safety [2, 26–28].

#### The influence of traditional medicine on delivery

Amongst the complications of TM ingestion during labor, we observed a significant risk of tachysystole (p = 0.008). Although we did not precisely identify which TM had this effect, this may infer that TM have a uterotonic property. As for honey, previous studies [23] revealed that it contains oestrogen, which could explain this uterotonic action. Although there is little available data on the effects of oestrogen on the human uterus, evidences from animal studies has it that oestrogen augments uterine contractility via increasing myometrial excitability, augmenting myometrial sensitivity to uterotonics such as oxytocin, and increasing the production and secretion of prostaglandins by foetal membranes [29–31]. Furthermore, honey is rich in inhibin which acts in synergy to procure stronger uterine contractions [23]. However, honey also has some toxicity related to the presence of toxic alkaloids or andrometoxin, a toxin from the nectar of the colchicine plant [23]. As such, this increases the risk of uterine atony in the exposed group. Another possible explanation is that the hyperkinetic uterus becomes fatigued after sometime of intense contractions and eventually failing to contract, leading to uterine atony. Lastly, we did not observe any difference in adverse foetal outcomes between the exposed and unexposed groups. Concurring findings were made by Holst et al. [15], who observed correlation between herbal medicine oral intake and neonatal complications.

### Conclusion

Overall, we found that about one parturient out of every four uses TM during labour. Some socio-demographic characteristics such as liberal union and tertiary level of education predispose pregnant women to TM ingestion during labour. TM oral intake during the peri-partum period was associated with vaginal bleeding prior to consultation, tachysystole, dystocic delivery and uterine atony.

## Limitations

The findings from the current study should be interpreted within the context of its limitations. These include the inability of 12.3% parturients to precisely identity what type of TM was taken prior to delivery or during labour (Table 1). Furthermore, TM oral intake during labour had no effect on foetal outcome perharbs due to the fact that the study was grossly underpowered to detect any significant differences in foetal outcomes such as stillbirth, hypoxia-ischemia, cerebral palsy, that are extremely rare events. Also, as the study population was drawn from only two referral maternities of Cameroon, this implies cautious generalization of the current findings. However, based on a large sample (n = 603) of well followed-up parturients, we have used robust statistical methods to contribute data on the contemporary scarcity of evidence the effects of peri-partum ingestion of TM on the maternal and foetal outcomes in the tropics. These findings could help guide the direction of future research into the safety and potential benefits of peripartum TM use, as well as serving as a preliminary reference for counselling pregnant women and obstetricians on TM use in obstetrics.

## Abbreviation

TM: traditional medicine
